# Development of calcium phosphate cement for the augmentation of traumatically fractured porcine specimens using vertebroplasty

**DOI:** 10.1016/j.jbiomech.2012.11.036

**Published:** 2013-02-22

**Authors:** Sami M. Tarsuslugil, Rochelle M. O’Hara, Nicholas J. Dunne, Fraser J. Buchanan, John F. Orr, David C. Barton, Ruth K. Wilcox

**Affiliations:** a School of Mechanical Engineering, University of Leeds, Leeds LS2 9JT, UK; b School of Mechanical and Aerospace Engineering, Queen's University Belfast, BT9 5AH, UK

**Keywords:** Spine, Trauma, Vertebroplasty, In-vitro, Biomechanical, Bone

## Abstract

The study aim was to develop and apply an experimental technique to determine the biomechanical effect of polymethylmethacrylate (PMMA) and calcium phosphate (CaP) cement on the stiffness and strength of augmented vertebrae following traumatic fracture. Twelve burst type fractures were generated in porcine three-vertebra segments. The specimens were randomly split into two groups (*n*=6), imaged using microCT and tested under axial loading. The two groups of fractured specimens underwent a vertebroplasty procedure, one group was augmented with CaP cement designed and developed at Queen's University Belfast. The other group was augmented with PMMA cement (WHW Plastics, Hull, UK). The specimens were imaged and re-tested . An intact single vertebra specimen group (*n*=12) was also imaged and tested under axial loading. A significant decrease (*p*<0.01) was found between the stiffness of the fractured and intact groups, demonstrating that the fractures generated were sufficiently severe, to adversely affect mechanical behaviour. Significant increase (*p*<0.01) in failure load was found for the specimen group augmented with the PMMA cement compared to the pre-augmentation group, conversely, no significant increase (*p*<0.01) was found in the failure load of the specimens augmented with CaP cement, this is attributed to the significantly (*p*<0.05) lower volume of CaP cement that was successfully injected into the fracture, compared to the PMMA cement. The effect of the percentage of cement fracture fill, cement modulus on the specimen stiffness and ultimate failure load could be investigated further by using the methods developed within this study to test a more injectable CaP cement.

## 1 Introduction

Every year, there are over 150,000 high-energy spinal fractures in the United States alone (Jones et al., 2011). Burst fractures are reported to account for about 15% of all spinal fractures (Denis, 1983) and usually result from a high rate of axial compression impact on the vertebrae within the thoracolumbar region. These fractures are commonly associated with motor vehicle accidents, falls from height or sports injuries and as such are prevalent in the younger population (Bensch et al., 2006; Briem et al., 2007).

Where surgical intervention is indicated for the treatment of traumatic fractures, the procedure is often highly invasive, involving posterior and/or anterior instrumentation (Verlaan et al., 2006). A limited number of case studies (Chen and Lee, 2004; Chen et al., 2004; Amoretti et al., 2005; Doody et al., 2009; Knavel et al., 2009) have reported the clinical use of vertebroplasty, involving the percutaneous injection of liquid polymethylmethacrylate (PMMA) bone cement directly into the fracture site, to treat burst fractures. These studies suggest that the treatment could reduce reliance on painkillers and increase mobility (Knavel et al., 2009).

Vertebroplasty has the advantage of being a less invasive procedure, but PMMA cement may not be the optimal material for the augmentation of traumatic burst fractures. It is not bioactive, so it is never incorporated into the bone. In addition, the cement experiences a high temperature exothermic reaction during polymerisation, which may lead to surrounding tissue necrosis (Eriksson et al., 1984). Calcium phosphate (CaP) cement has been considered as a potential alternative to PMMA because it allows the potential for bone in-growth, remodelling and the setting reaction occurs at body temperature. However, CaP cements have longer setting times and are characteristically brittle. There is a concern that the mechanical properties of these cements are not sufficient to provide vertebral stability and stiffness over the necessary duration for the cement to be resorbed by new bone and the fracture to heal. However, a limited number of clinical case studies have supported the use of CaP cement to augment traumatic vertebral fractures, with one reporting that complete bone union occurred 6 months after CaP augmentation of an osteoporotic burst fracture (Nakano et al., 2002). Whilst there is some clinical evidence of the potential of CaP cements for traumatic fracture augmentation, as yet, there appears to be little biomechanical evidence to support its use.

The aim of this study was to develop and apply an experimental technique to determine and compare the biomechanical effect of PMMA and CaP cements on the stiffness and failure load of augmented vertebrae following traumatic fracture.

## 2 Materials and methods

Porcine thoracolumbar (T7–L6) spinal tissue was used in the present study as a model for young human bone to represent the age of patients most prone to traumatic injury. The spines were obtained from an abattoir with all intervertebral discs and ligaments intact. The age of slaughter was between 6 and 8 months.

### 2.1 Intact vertebra tests

**Fig. 1:**
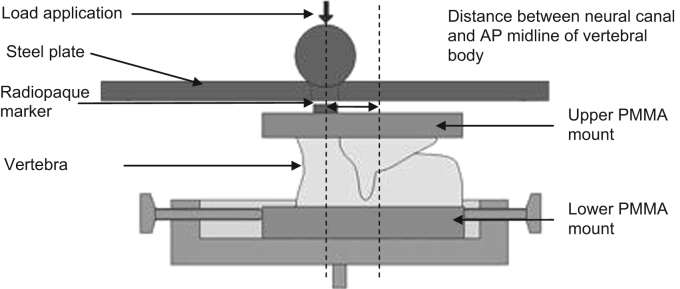
Loading scenario and radiopaque marker location for single vertebra specimens.

An initial series of tests were undertaken on intact vertebrae to establish the undamaged stiffness and failure load. Twelve single vertebrae were extracted from four spines. The posterior elements were trimmed but the inferior and superior articular processes were left intact. The specimens were mounted between two parallel PMMA endplates to ensure the specimen had flat surfaces on which to apply the axial load (Fig. 1).

The calculated stiffness of a vertebra has been shown to be highly sensitive to the location of the applied load (Wijayathunga et al., 2008). In this study, the centre of the vertebral body was chosen for the load application because it is reported to be the region most compromised following burst fracture (Magerl et al., 1994). The location was defined as the point along the centre line of the vertebra midway between the most anterior point of the neural arch and the most anterior point of the vertebral body. The position was measured using a pair of callipers and marked with a radiopaque disc which was affixed to the upper PMMA housing (Fig. 1). After the potting procedure, specimens were stored at a temperature of −18 °C until testing.

Following defrosting, the specimens were imaged using a micro-computer tomography (μCT) scanner (μCT80, Scanco, Switzerland) at a cubic voxel size of 74 μm. Each specimen was then tested under axial compression in a materials testing machine (Instron 3366, 10 kN Material Tester, Instron, UK) at a loading rate of 1 mm/min. Load was transmitted to the upper specimen cement mount via a steel ball bearing, housed within a stainless steel loading plate. The marker on the cement fitted into the steel housing to ensure the load was applied at the desired location (Fig. 1). The stiffness was determined as the largest gradient of the load–displacement curve obtained over a 0.6 mm displacement range, based on previous studies (Wijayathunga et al., 2008).

### 2.2 Burst fracture generation

A drop weight method was used in order to generate large open fractures and fragments of bone that are characteristic of traumatic fractures (Wilcox et al., 2003).

For this study, the porcine spines were sectioned into three-vertebra segments in order to maintain the interactions of the articular processes across the middle vertebra during compression. These interactions have been shown to be an important mechanism in the generation of burst fractures, causing areas of high strain to develop at the base of the pedicles (Wilcox et al., 2004). The inferior and superior vertebrae were set in PMMA endplates ensuring flat surfaces on which to apply the impact load.

The specimens were fixed into a steel housing and an impact load of known mass was dropped down a vertical guide shaft from a predetermined height onto the cranial end of the segment. The height and mass of the impact load was varied for each spinal level based on a preliminary study designed to optimise the success rate of burst fracture occurrence, resulting in an impact energy range of 121–142  J (Table 1).

**Fig. 2:**
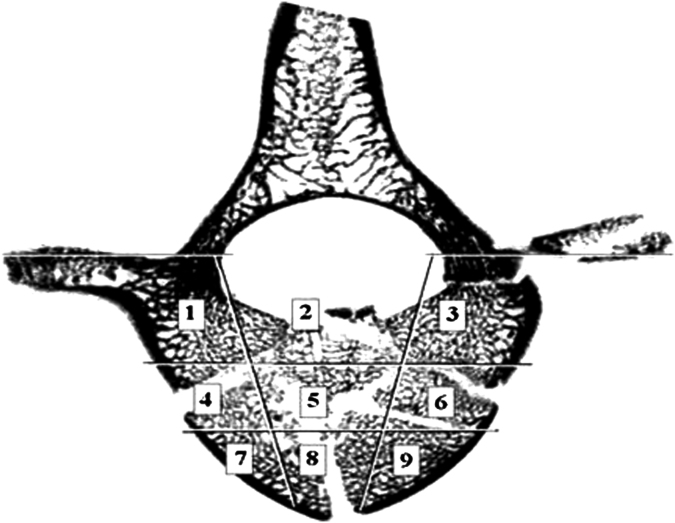
Vertebral body split into nine sections to aid in fracture diagnosis.

Following the fracture event, the middle vertebra of each segment was excised, mounted in cement plates and imaged using μCT. A scoring criteria adapted from the method employed by Panjabi et al. (1995) was used to determine the extent of fracture damage. Selections of μCT images of the fractured vertebral bodies were partitioned into nine sections (Fig. 2). Each section was assigned a score based on the interpretation of the extent of the injury that was observed, ranging from zero for no observable fracture to two for major fracture damage. Two regions of interest (coronal central, sections 4–6 and sagittal central, sections 2, 5 and 8) were defined to aid in the selection of the specimens for the vertebroplasty procedure, since these represented the region most compromised by the traumatic event and the intended sites for cement augmentation. Slices were chosen for analysis at approximately 2 mm intervals within the vertebral body region (every 30th slice), this yielded approximately ten slices required for analysis of each specimen. The vertebrae were deemed suitable for augmentation if the average scores for the two regions were over a threshold value.

**Fig. 3:**
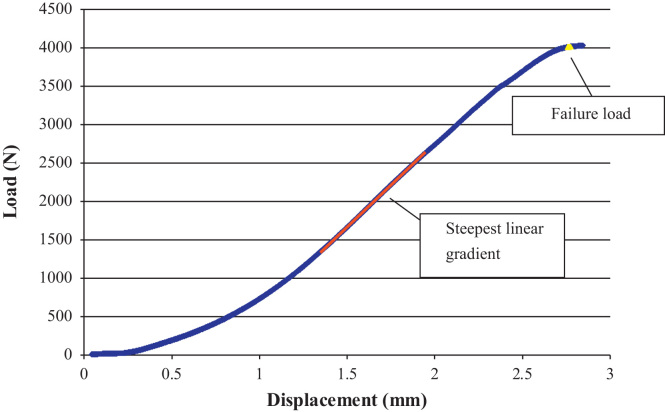
Load/displacement data obtained, typical for fractured specimens, the highlighted line indicates the steepest gradient of the graph for 0.6 mm of crosshead travel.

Twenty-four segments were initially excised from six porcine spines and, of these, 12 vertebrae yielded a fracture judged suitable for cement augmentation. The fractured specimens were randomly split into two groups (*n*=6) then imaged using μCT. Specimens were tested under static axial loading conditions using the same method as described for the intact vertebrae. All of the specimens tested exhibited regions of elasticity (Fig. 3), despite the traumatic injury that had preceded the axial compression. Once the specimens had been seen to reach a maximum load, testing was halted to minimise any further damage. A vertebroplasty procedure was then performed on all the specimens. One group was augmented with CaP cement designed and developed at Queen's University of Belfast (O’Hara et al., 2010), with a liquid to powder ratio of 0.35 mL/g, (compressive modulus, *E*_comp_=1.01 GPa). The main powder component used was alpha tri-calcium phosphate (α-TCP; Jack et al., 2008) and the liquid component was 4 wt% aqueous di-sodium hydrogen phosphate solution (Na_2_HPO_4_). The other group was augmented with laboratory grade PMMA cement (WHW Plastics, Hull, UK) liquid to powder ratio (w/w) of 5:3 (*E*_comp_=1.035 GPa).

### 2.3 Vertebroplasty method

All of the specimens underwent a bi-pedicular vertebroplasty procedure. First, the position of the needle and insertion depth (≈20–25 mm) was estimated by observation of the corresponding μCT scans that were taken after specimen fracture. Holes (diameter 3.5 mm) were then drilled at the appropriate positions to guide needle insertion.

Augmentation of the vertebrae using CaP cement was carried out as follows. Two 4-g batches of the appropriate cement were created. Following mixing, the cement was injected using 10 G needles until resistance prevented further extrusion. The needles were then sequentially retracted by approximately 5 mm and further injection was undertaken until all the cement had been injected or the resistance due to filter pressing in either the syringe nozzle or trabecular bone prevented any further extrusion. The specimens were stored for 5 days submerged in 0.03 wt% sodium azide Ringer's solution at 37 °C to enable the CaP to fully set. In this case, sodium azide was used to preserve the tissue for the duration of cement setting. A separate study was carried out to assess the effect of the sodium azide solution on the stiffness and ultimate failure load of both the porcine vertebrae and CaP cements, and it was found that the changes in mechanical properties were negligible at the low concentrations used (Tarsuslugil, 2011).

The injection of PMMA cement was achieved using 13 G needles. Two 4-g batches of cement were prepared and injected until the syringes were emptied, leakage into the vertebral canal was evident or no more cement could be extruded. The cement was left to set for approximately 40 min.

Following augmentation, all the specimens were μCT imaged and tested again under axial compression using the same conditions as were used for the intact specimens.

The μCT images of the pre- and post-augmentation specimens were segmented to identify the cement and fracture void regions using image analysis software (ScanIP, Simpleware Ltd., Exeter, UK). The volume of cement was calculated as a percentage fill of the total fracture volume for each specimen.

The stiffness and failure load values between the intact, fractured and two augmented groups of specimens were compared using a one-way analysis of variance (ANOVA, *α=*0.01) and post-hoc tests (Tukey–Kramer test, (*α=*0.01) for stiffness and Dunnett's test (*α=*0.01) for failure load). A *t*-test was used to compare the percentage fracture fill of the two cements injected (*α=*0.05).

## 3 Results

**Fig. 4:**
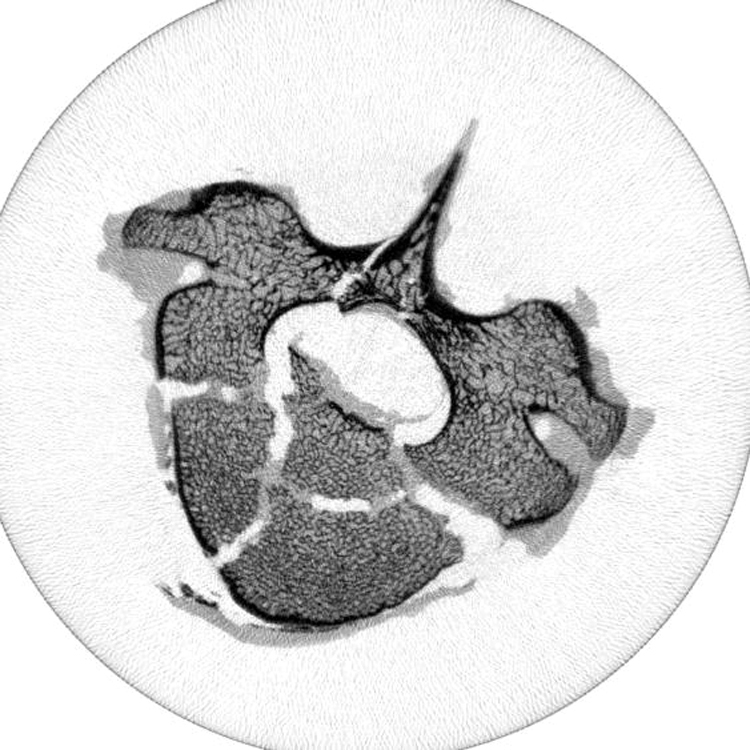
MicroCT transverse image of a typical specimen following fracture showing characteristic traumatic fracture pattern.

The drop weight method generated traumatic fractures with similar large fragments and fracture spaces to those observed in human bone, as shown in Fig. 4.

**Fig. 5:**
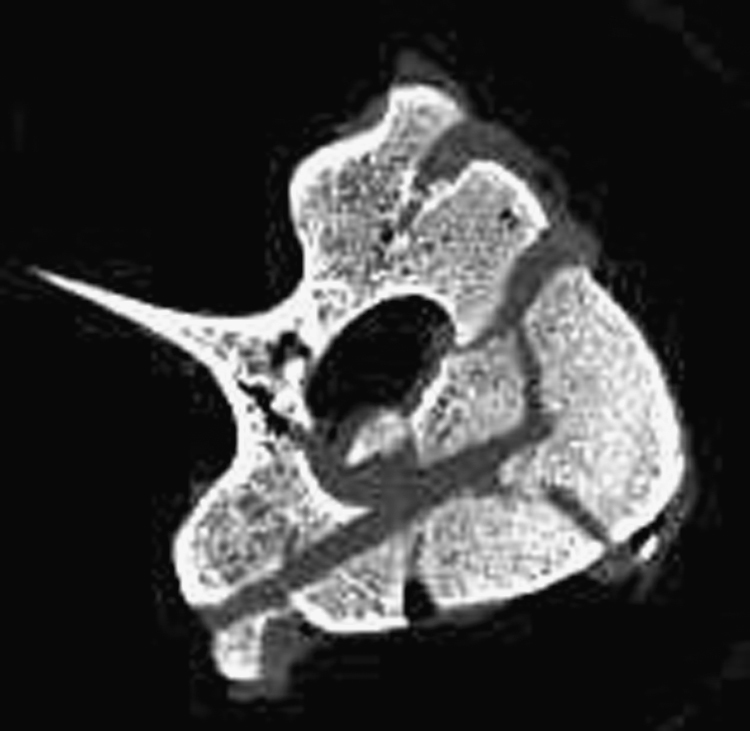
Evidence of PMMA bone cement leaking into vertebral canal.

Cement augmentation was successfully undertaken on all of the specimens, although the volume of cement injected varied between the cement types used. The CaP cement proved more difficult to inject due to its higher viscosity. From inspection of the syringe barrels immediately after the procedure, there was evidence of filter pressing and a thick layer of cement sediment was present, which had not been extruded from the syringe. From the analysis of the μCT images, the mean proportion of the fracture voids filled with this cement was 27% (SD±13%). The PMMA was comparably easier to inject and to control the volume of injection. Subsequently the mean percentage for fracture filled with cement was significantly higher 53% (SD±6%; *p*<0.05). However, as a possible consequence of its lower viscosity, the cement was more prone to leaking through the fracture fissures into the neural canal (Fig. 5). From observation of the μCT image data there appeared to be little evidence of cement penetration into the trabecular bone for either of the cements tested.

**Fig. 6:**
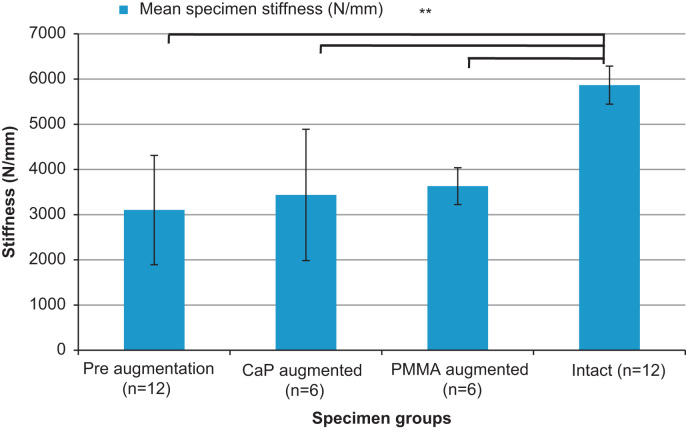
Comparison of mean of specimen stiffness (±SD) for intact, pre- and post-augmentation using the different cement types (⁎⁎denotes significant difference *p*<0.01).

From the mechanical testing, it was found that the fractured specimens had a significantly lower mean stiffness than the intact specimen group (*p*<0.01). The mean stiffness values of the augmented sets of specimens were also all found to be significantly lower than those of the intact specimens (*p*<0.01), indicating that neither of the cements injected were able to fully restore mean stiffness to the intact specimen level. Furthermore, neither of the cements injected were found to increase the mean specimen stiffness significantly, when compared to the fractured specimens (*p*<0.01; Fig. 6).

**Fig. 7:**
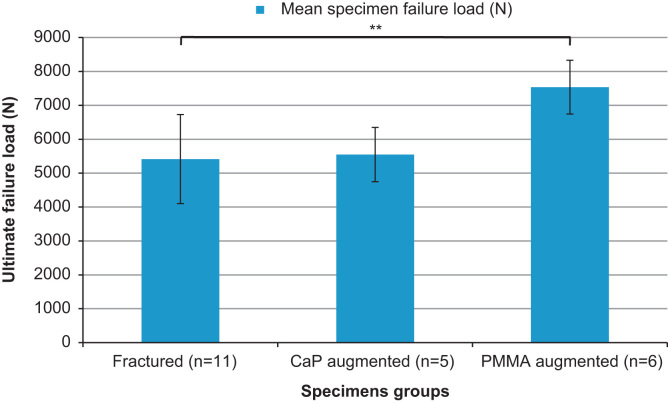
Comparison of mean of specimen ultimate failure load (±SD) pre- and post-augmentation using the different cement types (^⁎⁎^denotes significant difference *p*<0.01).

A significant increase in failure load was found for the specimen set augmented with PMMA cement when compared to their pre-augmentation values (*p*<0.01). The failure load of the specimens injected with CaP cement did not differ significantly from the pre-augmented values (*p*<0.01). The failure loads for the different specimen sets are shown in Fig. 7.

**Fig. 8:**
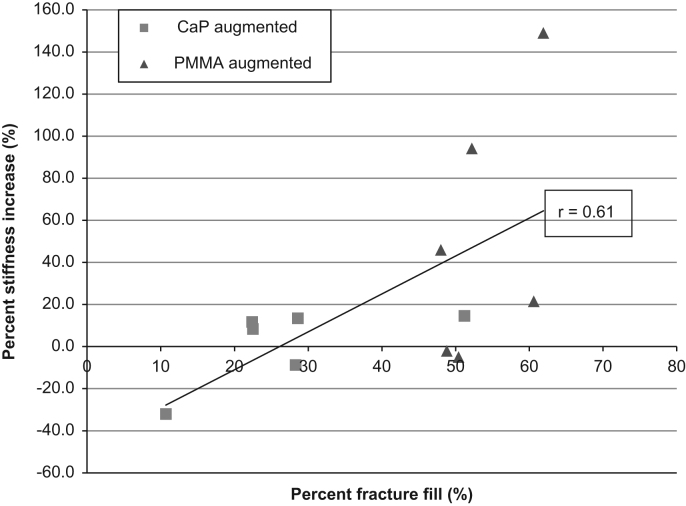
Relationship between percentage fracture fill and percentage stiffness increase for all cements.

The relationship between percentage fracture fill for both cements and percentage stiffness increase for each specimen is displayed in Fig. 8. From observation of the data it appears that there is a broad trend towards higher stiffness values for higher cement fills, Pearson's correlation coefficient, *r*(10)=0.61, *p*<0.05. The modulus of the CaP cement is comparable to that of the PMMA cement used (1.01 GPa and 1.035 GPa, respectively), which implies that the cause of the trend exhibited is not entirely due to the modulus difference between the cements injected.

## 4 Discussion

The use of vertebroplasty in the treatment of traumatic spinal fractures has received less attention from biomechanical research groups than its application in osteoporotic fracture management. As the number of clinical case studies increases, there is a need to increase the understanding of the biomechanical effects of vertebroplasty on the traumatically injured spine and to develop pre-clinical models to enable such treatments to be evaluated in the laboratory. The aim of this study was to develop such an experimental model and to use it to compare between PMMA and CaP cement as the augmentation material. Whilst there were inevitably variations in the specimens and fractures produced, the relatively small standard deviations in specimen stiffness and failure loads within groups, along with the significant difference in stiffness seen pre- and post-fracture, indicate that the fracture generation and screening methods did provide a suitable model for comparative cement studies.

The PMMA cement increased the stiffness and ultimate failure load by a greater margin than the CaP cement despite the similar compressive moduli of the two materials. The PMMA cement demonstrated the lowest viscosity on injection, hence, penetration into the fracture gaps was the most successful. From analysis of the μCT image data, there appeared to be little evidence of cement penetration into the trabecular structure and cement flow was governed by the location and distribution of the fractures present. The low viscosity of the cement increased the risk of the cement leaking though the fracture gaps. The CaP cement proved to be more difficult to inject even through the larger diameter 10 G needles, resulting in the occurrence of filter pressing. This is the separation of the liquid from the cement suspension (de-mixing), and occurs if the pressure required to extrude the cement is higher than the pressure required to filter the liquid through the cement powder (Bohner and Baroud, 2005). Filter pressing can result in very low viscosity cement, predominantly liquid, being injected and a thick ‘cake’ of cement sediment remaining in the syringe barrel, as was observed in this study. Consequently the percentage of the fracture gap filled with cement was significantly lower than the PMMA cement (*p*<0.05). The CaP cement augmentation presented very little improvement in failure load and no improvement in stiffness compared to the pre-augmented case.

These findings indicate that the cement viscosity and resulting penetration of the cement into the fracture volume has a prominent influence on the restoration of vertebral stability.

When comparing the post-augmented stiffness to the intact vertebra stiffness results (Fig. 6), it is clear that despite some improvement, even the most successful augmentation cement did not restore the stiffness to the values obtained pre-fracture . In terms of the failure loads, presented in Fig. 7, the values could not be compared directly the mean failure load for the intact specimens due to limitations in the load cell used (limit of 9.8 kN). The mean value of ultimate failure load for the specimens augmented using PMMA cement was found to be 7.5 kN, which suggests that despite the significant improvement over the pre-fracture level, the ultimate failure load for the augmented specimens were not fully restored to their intact levels. Another limitation is the use of a porcine model for human spinal tissue. Many studies have analysed or compared the two tissue types (Teo et al., 2006a, 2006b; Dath et al., 2007; Busscher et al., 2010) concluding that porcine vertebral bone is a reasonable model for human tissue. However, it was clear from the present study that the trabecular structure in the porcine vertebrae was more densely packed than that of the available human cadaveric tissue, which is often from a much older cohort than the patients that are typically susceptible to traumatic burst fracture (Bensch et al., 2006; Briem et al., 2007). While it is likely that the porosity of the porcine bone limited the penetration of the cements into the trabecular structure, this model effectively represents the worst case scenario for the injection of the cement, which is the augmentation of large open fractures within densely packed trabeculae. When considering the causes of spinal burst fracture and the mean age of the typical patient presented, the porcine model was therefore deemed acceptable as a representation of young human tissue.

In the case of the PMMA cements, these are important findings because it has no capacity to be resorbed into the bone and hence, the stiffness and strength increases brought about by the augmentation are likely to be at their maximum immediately after the cement has finished setting. In contrast, the CaP cement has the potential to allow fracture healing to take place, however, the CaP cement tested in the present study was limited in its capability to restore initial vertebral stiffness and failure load, by its inability to be successfully delivered via a cannulated needle. Further investigation into development of a more injectable CaP variation may improve the performance of the cement at restoring the vertebral stiffness and strength of traumatically fractured vertebra.

The principal method for improving the injectability of a CaP cement is by increasing its liquid to powder ratio (Bohner and Baroud, 2005), an undesirable trade-off for this, is often a reduction in the modulus (O’Hara et al., 2010). However, a number of studies have investigated different methods of improving the delivery of the CaP cements. For example, Bohner and Baroud (2005) concluded that the injectability could be improved by replacing the mixing liquid with a hydrogel. This method is thought to increase injectability by reducing the coefficient of friction between the CaP grains by introducing a polymer between them. Another study investigated the use of ultrasonication (Habib et al., 2012), whereby acoustic energy is used to agitate the suspended CaP cement particles during injection, this technique has been shown to significantly increase the injectability of the cement without de-mixing occurring. These methods could be investigated and incorporated in future studies. In addition the effect of the percentage of cement fracture fill and cement modulus on the specimen stiffness and ultimate failure load require further investigation. This could be achieved using the techniques developed within this study to test the different methods of injection and more injectable CaP cements, to determine if this would improve the stiffness and ultimate failure load of fractured vertebrae, to levels similar to values obtained when augmenting using the PMMA cement. This could also be investigated using computational methods, which would allow parameters such as cement modulus and percentage of fracture fill to be sequentially altered. Further studies are now underway to develop such models using the experimental data for validation. Whilst this study presented a static loading model, further work is also required to examine the performance of cements under fatigue conditions. Such studies are required to more fully understand, and ultimately optimise, this procedure before it is more widely introduced into clinic.

## Conflict of interest statement

There are no conflicts of interest within this study.

## Figures and Tables

**Table 1:** Drop heights and masses used to produce consistent burst fractures.

**Vertebral level**	**Drop height (m)**	**Drop mass (kg)**
T8–T9	1.40	9.05
T11–T12	1.55	9.05
L1–L2	1.60	9.05
L4–L5	1.20	10.28
